# Assessing the biobehavioral effects of ultramicronized-palmitoylethanolamide monotherapy in autistic adults with different severity levels: a report of two cases

**DOI:** 10.3389/fpsyt.2024.1463849

**Published:** 2024-10-22

**Authors:** Riccardo Bortoletto, Fabiana Piscitelli, Marta Basaldella, Claudia Scipioni, Carla Comacchio, Roberta Fiorino, Stefano Fornasaro, Pierluigi Barbieri, Daniele Pagliaro, Orietta Sepulcri, Martina Fabris, Francesco Curcio, Matteo Balestrieri, Marco Colizzi

**Affiliations:** ^1^ Unit of Psychiatry, Department of Medicine (DMED), University of Udine, Udine, Italy; ^2^ Institute of Biomolecular Chemistry, National Research Council (CNR), Pozzuoli, Italy; ^3^ Department of Medicine (DMED), University of Udine, Udine, Italy; ^4^ Institute of Clinical Pathology, Friuli Centrale Health University Authority (ASUFC), Udine, Italy; ^5^ Department of Chemical and Pharmaceutical Sciences, University of Trieste, Trieste, Italy; ^6^ Unit of Psychiatry, Friuli Centrale Health University Authority (ASUFC), Udine, Italy; ^7^ Department of Psychosis Studies, Institute of Psychiatry, Psychology and Neuroscience, King’s College London, London, United Kingdom

**Keywords:** neurodevelopmental disorders, peroxisome proliferator activated receptor alpha, glutamate signaling, nutraceutical, supplementary food, cannabinoids, entourage effect

## Abstract

Despite promise of its supplementation as both monotherapy and add-on treatment in autism spectrum disorder (ASD), the biobehavioral effects of Palmitoylethanolamide (PEA) in autistic adults have never been explored so far. We discussed the cases of two autistic adults with different degrees of severity (level 1 and level 2) presenting with symptoms of psychic distress, who were treated with ultramicronized-PEA (um-PEA) 600 mg/day monotherapy for a sustained period of 4 months. The level 1 autistic patient showed improved depressive symptoms and social engagement at a 12-week follow-up, in parallel to a tendency toward reduced inflammatory response and enhanced endocannabinoid (eCB) signaling, partially relapsing after um-PEA discontinuation at four months. Opposedly, the level 2 autistic patient exhibited a generally stable psychosocial functioning for the initial 12 weeks, consistent with basically unchanged immune and eCBs levels, abruptly deteriorating and leading to antipsychotic initiation afterwards. No significant side effects were reported in both cases during the observation period. The two cases suggest that um-PEA could be an effective option for the treatment of psychic distress in level 1 autistic adults, warranting further investigation of its age- and level-specificity and of the biological underpinnings of its therapeutic effect in ASD.

## 1 Introduction

Autism Spectrum Disorder (ASD) is a complex multifactorial neurodevelopmental condition entailing difficulties in social interaction and restricted/repetitive behaviors and interests, presenting with varying degrees of severity (1–3). Autistic individuals often encounter numerous psychosocial barriers and challenges starting from early childhood (4–6), leading to limited social integration, poor job prospects, and a high incidence of comorbid psychic distress during adulthood (7–11). Detecting and intervening early on symptoms of psychic distress in autistic adults is therefore crucial to maintaining their quality of life, although mixed evidence regarding whether they may benefit from conventional psychotropic medications (12, 13) warrants the need to research into novel therapeutical targets. Despite being largely debated (14), a comprehensive understanding of the neurophysiological mechanisms underlying ASD is still hard to define. In recent years, growing evidence has converged toward immune dysregulation as a key contributor to the emergence and persistence of ASD traits, either systemically (15–19), or within the central nervous system (CNS), where it primarily occurs as glutamate excitotoxicity (20–23). Concurrently, over the past decades the endocannabinoid (eCB) system has drawn increasing attention for its ability to modulate neuroinflammation and glutamatergic signaling to dopaminergic neurons via the cannabinoid receptors (i.e., CB_1_ and CB_2_ receptors) interaction with both exogenous (i.e., phytocannabinoids) and endogenous (i.e., eCBs) ligands (24–26), with growing but sparse evidence of potential biobehavioral and therapeutic implications for ASD (27–29). Along with the major eCBs anandamide (AEA) and 2-arachidoylgylcerol (2-AG), several other bioactive mediators have been discovered, including the *N*-acylethanolamines (NAEs) palmitoylethanolamide (PEA) and oleoylethanolamide (OEA), monoacylglycerols, and other *N*-acylaminoacids/neurotransmitters, all of which are part of the expanded eCB system, known as the endocannabinoidome (eCBome) (30). In particular, PEA has been increasingly studied for its immunomodulatory properties, acting through an endocannabinoid-like mechanism both in the peripheral nervous system and in the CNS (31, 32), with biobehavioral correlates in several neurological and mental health diseases, including epilepsy (33), multiple sclerosis (34), cognitive decline (35), major depressive disorder (36), and psychosis (37). PEA presents a multi-faceted mechanism of action. First it acts as a direct agonist of the Peroxisome Proliferator Activated Receptor-α (PPAR-α) and of the G Protein-coupled Receptor 55 (GPR55), second as an allosteric modulator of the Transient Receptor Potential Vanilloid 1 (TRPV1), and third as an inhibitor of the Fatty-Acid Amide Hydrolase (FAAH) and a stimulator of the Diacylglycerol Lipase (DAGL), thus increasing the endogenous availability of AEA and 2-AG (31, 32). In addition to being naturally produced by several human cells and tissues in response to actual or potential damage, PEA can also be supplemented exogenously. In its ultra-micronized form (um-PEA), it shows enhanced bioavailability by more effectively penetrating the CNS (38).

The biobehavioral role of PEA in ASD has been recently interrogated, particularly for its therapeutic potential to target the immune-glutamatergic pathway via the eCB system modulation (39). While evidence has been reported on the beneficial effects of PEA supplementation, either as monotherapy or as add-on to treatment as usual with a safe and tolerable profile, on the core symptoms of autistic children and adolescents (40–42), to the best of our knowledge it has not yet been investigated among autistic adults.

Here, we present the cases of two autistic adults treated with um-PEA as monotherapy for symptoms of psychic distress over a sustained period of 4 months. To better account for heterogeneous effects of um-PEA across the autism spectrum (1), we describe the cases of patients differing in their levels of support needs and presence of comorbid intellectual disability.

## 2 Case reports

**Figure 1:**
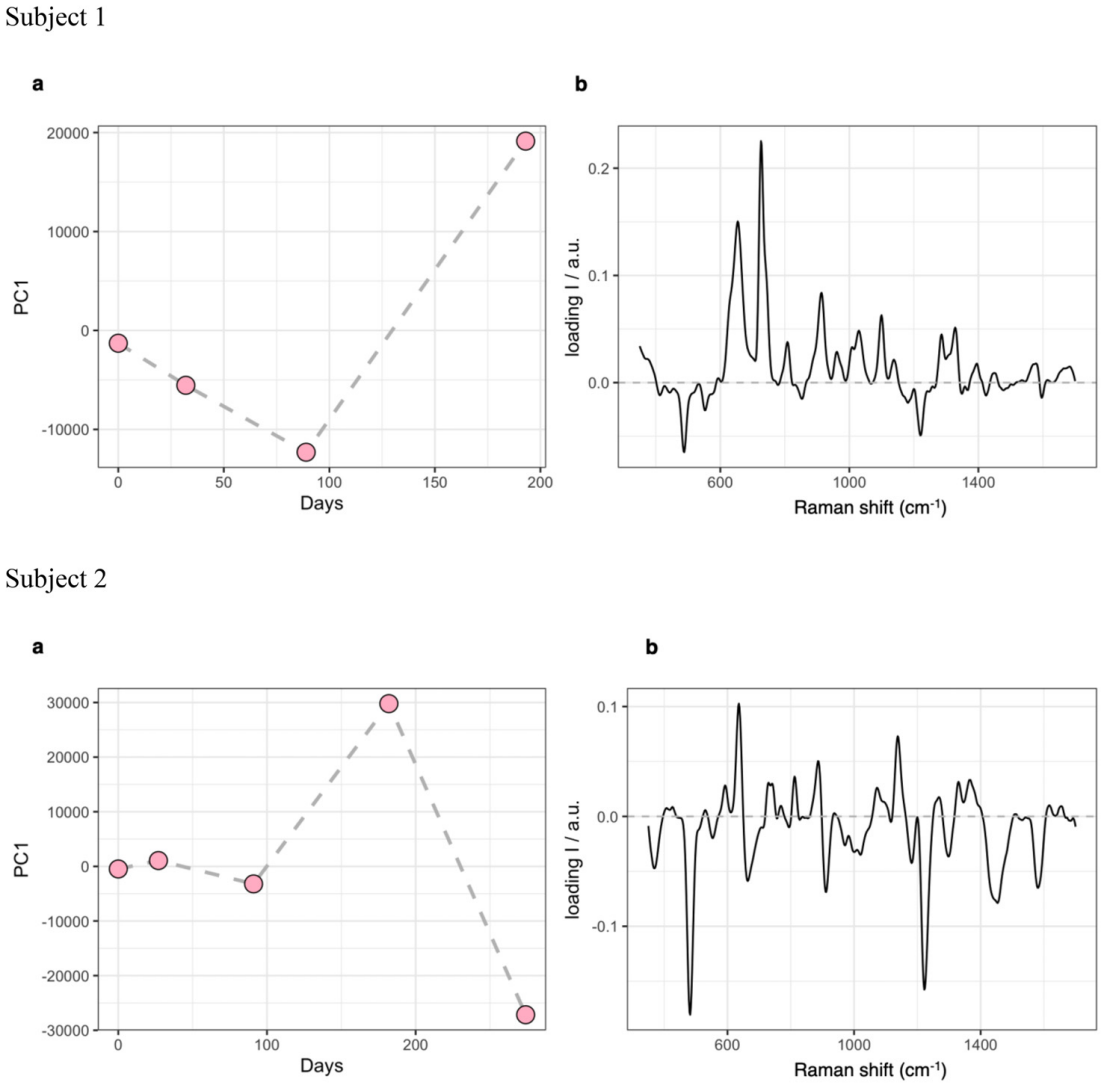
PCA results for the analysis SERS dataset. **(A)**, evolution of the PC1 score profile over time. **(B)**, spectral loadings for PC1. PCA, Principal Components Analysis; SERS, Surface-Enhanced Raman Scattering; PC1, Principal Component 1.

Both patients were help-seeking women presenting with symptoms of psychic distress to the Unit of Psychiatry at the University Hospital of Udine, where they were diagnosed with ASD in adulthood according to the Diagnostic and Statistical Manual of Mental Disorders—Fifth Edition (DSM-5) (1). Baseline evaluations were carried out to define neurodevelopmental characteristics [the Ritvo Autism Asperger Diagnostic Scale-Revised (RAADS-R) (43), the Autism Quotient (AQ) (44), the Empathy Quotient (EQ) (45), the Camouflaging Autistic Traits Questionnaire (CAT-Q) (46), the Wechsler Adult Intelligence Scale—Fourth Edition (WAIS-IV) (47), the World Health Organization Disability Assessment Schedule 2.0 (WHODAS 2.0; 36 items, interviewer-administered) (48)] and to assess possible neuropsychiatric comorbidities [the Structured Clinical Interview for DSM-5 Disorders, Clinician Version (SCID-CV) (49), the Structured Clinical Interview for DSM-5 Disorders, Personality Disorders Version (SCID-PD) (50), the Esame Neuropsicologico Breve-2 (ENB-2, meaning Brief Neuropsychological Examination 2nd Edition in Italian) (51)] (**Table 1**). The patients were approached by trained investigators to initiate um-PEA monotherapy, as the first two consecutive autistic individuals with heterogeneous ASD severity levels consenting to an internal pilot trial approved by the Department of Medicine (DMED) at the University of Udine (Institutional Review Board: 146/2024) in May 2024. They were proposed to undergo sustained daily treatment with oral um-PEA (600 mg/day with breakfast, tablet form, Normast^®^), to assess the feasibility of completing a 12-week follow-up. The medication was dispensed by qualified physicians in units of 60 tablets at each timepoint. Clinical progress was evaluated at baseline, 4 weeks, and 12 weeks using (i) the Symptom Checklist-90-Revised (SCL-90-R) (52) to assess um-PEA effect on levels of psychic distress (**Table 2A**), (ii) the Hospital Anxiety and Depression Scale (HADS) (53) to assess um-PEA effect on anxiety and depressive symptoms (**Table 2B**), and (iii) the WHODAS 2.0 (48) to assess um-PEA effect on adaptive functioning (**Table 2C**). The occurrence of adverse effects was quantified using the UKU Side Effect Rating Scale (54) (**Table 2D**). After completing the initial 12-week phase, the patients were invited to enter an extension phase follow-up to assess the clinical stability of the treatment for a maximum observation period of 36 weeks. At each timepoint, patients underwent blood analyses, including hematological, biochemical, inflammatory, and neuroaxonal impairment markers (**Table 3**). Specifically, a large panel of cytokines and chemokines was analyzed on available serum samples using customized multiplex immunoenzymatic assays (Ella instrument, Bio-Techne, USA). Reference ranges suggested by the manufacturer were validated in our population by indirect methods. Adjunctive serum samples were collected to measure the biochemical reaction pathways resulting from um-PEA supplementation using Surface-Enhanced Raman Scattering (SERS) technique (55) (**Figure 1**; **Supplementary Material**) and to measure endocannabinoidome (eCBome) mediators using liquid chromatography-mass spectrometry (LC-MS) (56, 57) (**Table 3**). Blood samples collection was performed at 8 in the morning, after fasting for approximately 12 hours, before um-PEA daily intake.

**Table 1:** Sociodemographic and clinical characteristics.

	Subject 1	Subject 2
Sex	F	F
Age at the first assessment	35,0	39,9
Schooling	Post-secondary education	Secondary school
Housing situation	With parents	With parents
Occupation - Economic maintenance	Employed	Employed
Diagnosis	ASD level 1	ASD level 2, moderate intellectual disability
RAADS-R	Tot: 94; S1: 50; S2: 17; S3: 6; S4: 221	Tot: 84; S1: 53; S2: 16; S3: 8; S4: 7
AQ	Tot: 23; AQ-1: 7; AQ-2: 7; AQ-3: 5; AQ-4: 3; AQ-5: 1	Tot: 29; AQ-1: 7; AQ-2: 6; AQ-3: 3; AQ-4: 5; AQ-5: 8
EQ	Tot: 46	Tot: 33
CAT-Q	Tot:140; C: 51; M: 42; A: 47	Tot: 96; C: 28; M: 39; A: 29
WAIS-IV	FS-IQ: 106; VCI: 106; PRI: 108; WMI: 86; PSI: 117	FS-IQ: 72; VCI: 69; PRI: 79; WMI: 83; PSI: 86
WHODAS 2.0	Overall score: 25.50%	Overall score: 34.77%
SCID-5-CV	Negative	Intellectual disability (moderate severity), Autism Spectrum Disorder Level 2, and Anorexia Nervosa (mild, in partial remission, type with restriction)
SCID-5-PD	Negative	Negative
ENB-2	DS: 5 (normal); IPM: 20 (normal); DPM: 22 (normal); IM (10): 9 (normal); IM (30): 9 (normal); SA: 19.75 (normal); AS: 65.33 (normal); PF: 19 (normal); VC: 5 (normal); AA: 5 (normal); CE: 4 (borderline); VRD: 43 (normal); VSA (Copying a drawing, Spontaneous drawing): 2, 2 (normal); SEF (Clock Test): 10 (normal); Praxis: 6 (normal); OCI: 91 (normal)	DS: 8 (normal); IPM: 3 (below the norm); DPM: 0 (below the norm); IM (10): 2 (below the norm); IM (30): 8 (normal); SA: 34.85 (normal); AS: 191 (below the norm); PF: 7.7 (below the norm); VC: 4.5 (below the norm); AA: 2 (below the norm); CE: 3 (below the norm); VRD: 17 (below the norm); VSA (Copying a drawing, Spontaneous drawing): 2, 2 (normal); SEF (Clock Test): 10 (normal); Praxis: 5 (below the norm); OCI: 59 (below the norm)

F, female; ASD, Autism Spectrum Disorder; RAADS-R, Ritvo Autism Asperger Diagnostic Scale–Revised; S1, Social Interaction subscale; S2, Circumscribed Interests subscale; S3, Language subscale; S4, Sensory-Motor subscale; AQ, Autism Quotient; AQ-1, Social Skill subscale; AQ-2, Attention Switching subscale; AQ-3, Attention to Detail subscale; AQ-4: Communication subscale; AQ-5: Imagination subscale; EQ, Empathy Quotient; CAT-Q: Camouflaging Autistic Traits Questionnaire; C, Compensation subscale; M: Masking subscale; A, Assimilation subscale; WAIS-IV: Wechsler Adult Intelligence Scale- Fourth Edition; FS-IQ, Full Scale Intelligence Quotient; VCI, Verbal Comprehension Index; PRI, Perceptual Reasoning Index; WMI, Working Memory Index; PSI, Processing Speed Index; WHODAS 2.0, World Health Organization Disability Assessment Schedule 2.0; SCID-5-CV, Structured Clinical Interview for DSM-5- Clinical Version; SCID-5-PD, Structured Clinical Interview for DSM-5 Personality Disorder; ENB-2, Esame Neuropsicologico Breve-2 (meaning Brief Neuropsychological Examination 2nd Edition in Italian); DS, Digit Span; IPM, Immediate Prose Memory; DPM, Delayed Prose Memory; IM, Interference Memory; SA, Selective Attention; AS, Attention Shifting; PF, Phonemic Fluency; VC, Verbal Comprehension; AA, Abstraction Abilities; CE, Cognitive Estimations; VRD, Visual Recognition and Discrimination; VSA, Visuo-Spatial Abilities; SEF, Spatial and Executive Functioning; OCI, Overall Cognitive Index.

**Table 2:** Clinical assessments: (A) The Symptom Checklist 90-Revised.

Case	Timepoint	SCL-90-R						
**Subject 1**		GSI	PST	PSDI	SOM	O-C	I-S	
	Baseline	2.76	1.00	–	1.42	2.70	4.00	
	Week 12	2.17	1.00	–	1.17	2.40	2.67	
	Week 24	2.04	1.00	–	1.08	1.10	3.22	
		DEP	ANX	HOS	PHOB	PAR	PSY	SLEEP
	Baseline	3.62	2.30	2.83	1.86	4.50	1.80	4.00
	Week 12	1.92	1.90	4.17	1.57	3.67	1.40	3.00
	Week 24	2.54	1.50	2.33	1.71	3.67	1.50	4.00
Case	Timepoint	SCL-90-R						
**Subject 2**		GSI	PST	PSDI	SOM	O-C	I-S	
	Baseline	1.10	1.00	–	1.00	1.40	1.11	
	Week 12	1.10	1.00	–	1.00	1.20	1.11	
	Week 36	1.50	1.00	–	1.17	3.20	1.44	
		DEP	ANX	HOS	PHOB	PAR	PSY	SLEEP
	Baseline	1.15	1.10	1.00	1.00	1.00	1.00	1.33
	Week 12	1.15	1.10	1.17	1.00	1.00	1.00	1.67
	Week 36	1.54	1.20	1.17	1.43	1.00	1.20	1.67

SCL-90-R, The Symptom Checklist 90-Revised; GSI, Global Severity Index; PST, Positive Symptom Total; PSDI, Positive Symptom Distress Index; SOM, Somatization subscale; O-C, Obsessive-Compulsive subscale; I-S, Interpersonal Sensitivity subscale; DEP, Depression subscale; ANX, Anxiety subscale; HOS, Anger-Hostility subscale; PHOB, Phobic-anxiety subscale; PAR, Paranoid ideation subscale; PSY, Psychoticism subscale; SLEEP, Sleep disturbances subscale.

**Table 2B:** The Hospital Anxiety and Depression Scale.

Case	Timepoint	HADS	
**Subject 1**		ANX	DEP
	Baseline	9	13
	Week 12	8	15
	Week 24	5	11
**Subject 2**		ANX	DEP
	Baseline	1	7
	Week 12	1	6
	Week 36	3	12

HADS, The Hospital Anxiety and Depression Scale; ANX, Anxiety score; DEP, Depression score.

**Table 2C:** The World Health Organization Disability Assessment Schedule 2.0.

Case	Timepoint	WHODAS-2.0						
**Subject 1**		D1	D2	D3	D4	D5	D6	Total
	Baseline	8	5	5	16	9	16	59
	Week 12	11	5	4	14	15	13	62
	Week 24	6	5	4	13	8	25	61
**Subject 2**		D1	D2	D3	D4	D5	D6	Total
	Baseline	9	5	4	10	8	15	51
	Week 12	11	5	8	21	11	12	68
	Week 36	9	5	5	17	13	10	61

WHODAS-2.0, The World Health Organization Disability Assessment Schedule 2.0; D1, Understanding and communicating domain; D2, Getting around domain; D3, Self-care domain; D4, Getting along with people domain; D5, Life-activities (Household and School/Work) domain; D6, Participation in society domain.

**Table 2D:** The UKU-Side Effect Rating Scale.

Case	Timepoint	UKU-SERS			
**Subject 1**		Total PSYCH	Total NEURO	Total AUTO	Total OTHER
	Baseline	11	5	4	12
	Week 4	2	0	4	1
	Week 12	9	1	3	3
	Week 24	8	0	1	3
**Subject 2**		Total PSYCH	Total NEURO	Total AUTO	Total OTHER
	Baseline	1	0	1	0
	Week 4	1	0	0	0
	Week 12	5	0	2	0
	Week 24	0	0	0	0
	Week 36	4	0	3	0

UKU-SERS, The UKU-Side Effect Rating Scale; PSYCH, Psychic side effects; NEURO, Neurologic side effects; AUTO, Autonomic side effects.

**Table 3:** Main hematological, biochemical, inflammatory, neuroaxonal impairment, and endocannabinoidome (eCBome) markers.

Case	Timepoint	WBC count (x10^3/µl)	Monocyte count (x10^3/µl)	N/L Ratio	SII Index ((N x PLT)/L Ratio)	ESR (mm/h)	CRP (mg/L)	Cortisol 8AM (nMol/L)
**Subject 1**	Baseline	6.07	0.52	1.48	508.73	7.00	0.24	529.00
	Week 4	6.04	0.59	1.36	479.97	9.00	n.a.	673.00
	Week 12	4.72	0.43	1.31	395.17	7.00	0.43	503.00
	Week 24	7.54	0.46	4.80	1574.4	6.00	0.02	347.00
**Subject 2**	Baseline	5.18	0.33	2.85	542.20	3.00	0.18	332.00
	Week 4	4.72	0.35	1.92	346.25	5.00	0.22	384.00
	Week 12	4.42	0.34	1.98	310.46	5.00	0.60	408.00
	Week 24	3.75	0.37	1.52	239.01	9.00	0.60	386.00
	Week 36	4.34	0.31	2.50	459.20	8.00	0.60	502.00

Case	Timepoint	IL-1β (pg/mL)	IL-6 (pg/mL)	IL-8 (pg/mL)	TNF-α (pg/mL)	IL-2Rα (pg/mL)	IL-10 (pg/mL)	IP10 (pg/mL)	IFN-γ (pg/mL)	CX3CL1 (pg/mL)
**Subject 1**	Baseline	0.20	2.00*	16.10	9.10	495.00	0.90	40.50	0.10	1192.00
	Week 4	0.30	2.00*	10.70	7.80	852.00	1.30	69.90	0.20	1111.00
	Week 12	0.00**	2.00*	51.10	7.20	776.00	1.30	74.00	0.30	1125.00
	Week 24	0.00**	2.00*	28.70	7.70	941.00	1.90	70.90	0.30	1110.00
**Subject 2**	Baseline	0.00**	2.00*	12.30	4.80	1155.00	1.80	79.80	0.30	1229.00
	Week 4	0.00**	2.00*	10.70	4.80	928.00	n.a.	101.00	0.30	1046.00
	Week 12	0.00**	2.00*	7.30	5.50	889.00	1.20	104.00	0.30	1117.00
	Week 24	0.00**	2.00*	7.40	6.60	1118.00	4.80	157.00	0.70	1379.00
	Week 36	0.00**	2.00*	9.10	4.80	1074.00	2.40	63.50	0.30	1054.00

Case	Timepoint	IL-15 (pg/mL)	Nf-L (pg/mL)	AEA (pmol/ml)	2-AG (pmol/ml)	PEA (pmol/ml)	OEA (pmol/ml)	DHA-EA (pmol/ml)	EPA-EA (pmol/ml)	2-DHG (pmol/ml)
**Subject 1**	Baseline	2.90	11.10	4.40	31.90	11.70	15.50	12.17	1.60	196.40
	Week 4	3.00	9.50	5.60	32.10	107.60	19.80	239.32	1.60	171.20
	Week 12	2.70	12.90	9.70	43.60	15.20	22.70	220.31	1.00	210.10
	Week 24	3.10	14.30	6.30	23.20	11.10	18.30	185.03	1.20	375.70
**Subject 2**	Baseline	2.30	8.20	6.50	28.80	30.80	29.10	14.16	1.60	123.00
	Week 4	1.90	8.80	3.20	26.60	8.10	7.00	210.35	1.20	197.50
	Week 12	2.10	15.30	6.60	28.80	10.60	17.60	229.26	1.40	162.70
	Week 24	2.00	12.40	2.30	16.20	7.00	5.50	212.69	1.10	1593.50
	Week 36	1.80	10.00	4.50	13.10	15.00	13.70	162.77	1.00	657.10

WBC, White blood cell; N/L, Neutrophil/lymphocyte; SII, Systemic immune-inflammation; ESR, Erythrocyte sedimentation rate; CRP, C-reactive protein; n.a., not assessed; AM, ante meridian; IL-1β Interleukin-1β; IL-6, Interleukin-6; *, real value; **, lower limit of detection range; IL-8, Interleukin-8; TNF-α, Tumor Necrosis Factor-α; IL-2Rα, Interleukin-2Rα; IL-10, Interleukin-10; IFN-γ, Interferon-γ; CX3CL1, Fractalkine; IL-15, Interleukin-15; Nf-L, Neurofilament-light chain; AEA, Anandamide; 2-AG, 2-arachidoylgylcerol; PEA, Palmitoylethanolamide; OEA, Oleoylethanolamide; DHA-EA, Docosahexaenoyl ethanolamide; EPA-EA, Eicosapentaenoyl ethanolamide; 2-DHG, Docosahexanoyl-glycerol.

A synthesis of the two cases is presented below and summarized in **Tables 1**–**3**, and **Figure 1.**

### 2.1 Subject 1

The patient is a 35-year-old woman, who was diagnosed in adulthood with ASD. She lives with her parents. Allegedly, her family history is negative for any neuropsychiatric conditions. Her developmental milestones were reached on time. She experienced slight difficulties in math during schoolyears, although no failures were reported. She finally obtained a degree in Cultural Heritage Conservation and currently works in a library. Premorbid social adjustment was described as subnormal, with tendency to rigidity, avoidance of social contacts, and difficulties to develop age-appropriate relationships. Signs of tactile, gustative, and auditory hypersensitivity were referred.

A clinical diagnosis of level 1 ASD was based on the DSM-5 criteria (1, 49) and supported by the RAADS-R (43) and the CAT-Q (46). The EQ test (45) displayed good empathic abilities. The AQ test (44) displayed subthreshold results. Her intelligence quotient (IQ) was within normal limits, as measured using the WAIS-IV (47). Further evaluation with the WHODAS 2.0 showed a level of daily functioning with mild impairment. Although by the time of her presentation to the Unit of Psychiatry outpatient service she exhibited long-standing depressive symptoms, irritability, low concentration, restrictive eating behaviors, and sleep disturbances, their severity was not sufficient to diagnose comorbid major psychiatric or personality disorders, as measured with the SCID-5-CV (49) and SCID-5-PD (50), respectively (**Table 1**). She was not on any psychotropic medications. In the past, the patient was prescribed sertraline 50 mg/day, perphenazine 2 mg/day, and gabapentin 900 mg/day to treat the aforementioned symptoms of psychic distress. However, these medications provided little benefit and resulted in the occurrence of unpleasant side effects, so that the patient was reluctant to initiate further conventional psychopharmacological treatments.

She then agreed to start supplementation monotherapy with um-PEA 600 mg/day for 12 weeks, resulting in improvement on the Global Severity Index (GSI) and on the somatization, obsessive-compulsive, interpersonal sensitivity, depression, anxiety, phobia, paranoia, psychoticism, and sleep subscales of the SCL-90-R (52) (**Table 2A**). Despite discontinuing um-PEA at 4 months, most beneficial effects persisted and were accompanied by improvements in the total anxiety and depression scores of the HADS (53) (**Table 2B**) when the patient was reassessed at Week 24. Conversely, the depression subscale of the SCL-90-R (52) (**Table 2A**) at Week 24 partially regressed compared to Week 12, along with the interpersonal sensitivity, phobia, psychoticism, and sleep subscales. Most domains of the WHODAS 2.0 (48) improved at Week 24 compared to the treatment initiation, although the total score of the scale remained nearly unchanged, possibly due to a sharp decline in the Participation in society domain from Week 12 to Week 24 (**Table 2C**). No serious side effects were reported throughout the observation period (54) (**Table 2D**).

At Week 12, several changes in blood and serum immune response biomarkers were observed compared to baseline, including decreased total white blood cell (WBC) count, neutrophil/lymphocyte ratio, monocyte count, systemic immune-inflammation (SII) index, cortisol at 8 ante meridiem (AM), Interleukin-1β (IL-1β, normal range <0.16 pg/mL), Tumor Necrosis Factor-α (TNF-α, normal range 7.80-12.20 pg/mL), Interleukin-15 (IL-15, normal range 1.90-2.60 pg/mL), and fractalkine (CX3CL1, normal range 892.00-1502.00 pg/mL), with some of these markers reversing to higher levels by Week 24 after um-PEA discontinuation. Conversely, increasing patterns in Interleukin-8 (IL-8, normal range 6.70-16.20 pg/mL), Interleukin-2Rα (IL-2Rα, normal range 440.00-1435.00 pg/mL), Interleukin-10 (IL-10, normal range 1.80-3.80 pg/mL), Interferon-γ (IFN-γ, normal range <0.99 pg/mL), Interferon-inducible Protein 10 (IP10, normal range 37.20-222.00 pg/mL), and C-reactive protein (CRP) were detected at Week 12 compared to baseline, mostly maintaining stable levels at Week 24 after um-PEA discontinuation. Erythrocyte sedimentation rate (ESR) and Interleukin-6 (IL-6, normal range <7.00 pg/mL) levels remained stable and within normal range for the entire observation period. Neurofilament-light chain (Nf-L, normal range 9.00-22.00 pg/mL) showed a tendency toward increase, although maintaining within normal limits (**Table 3**).

The Principal Components Analysis (PCA) of samples used for SERS spectroscopy indicated that the main molecular fingerprint was related to spectral bans commonly linked to serum levels of purine degradation products (at 654 and 726 cm^−1^) (58). These bands showed a significant decrease from baseline to Week 12, partially reflecting changes related to reduced inflammation, followed by a sharp increase by Week 24 (**Figure 1**).

Markers of eCBome modulation included elevated levels of serum AEA, 2-AG, OEA, PEA, docosahexaenoyl ethanolamide (DHA-EA), and 2-docosahexanoyl-glycerol (2-DHG) by Week 12 compared to baseline, with most levels subsiding after the discontinuation of um-PEA. Besides, levels of eicosapentaenoyl ethanolamide (EPA-EA) decreased at Week 12 but increased again by Week 24 (**Table 3**).

### 2.2 Subject 2

The patient is a 40-year-old woman, who was diagnosed in adulthood with ASD and comorbid moderate intellectual disability. She lives with her parents. The patient’s family history is negative for any neuropsychiatric conditions. She was born preterm (after eight months of gestation) out of her parents’ second pregnancy. At the time of her presentation to the Unit, no anamnestic data were available regarding postnatal electroencephalography (EEG) recordings or brain magnetic resonance imaging (MRI). She presented delayed expressive language development with need for one-year speech therapy when she was 3 years old. Allegedly, all other developmental milestones were reached on time. The patient has presented serious impairment in relationships with peers since early childhood, showing a tendency to self-isolation and difficulties to integrate herself within social groups. Her premorbid role functioning was marked by poor performance in mainstream school courses, ongoing challenges in generating and organizing homemaking tasks, and inability to maintain an independent job after high school. The latter difficulty was mainly due to struggles with manual activities and relating to customers. According to her mother, the patient faced all novelties with deep anxiety, driven by her extreme rigidity, which also affected her limited interests. Following poor performance evaluations at her first work placements when she was nineteen years old, the patient displayed initial signs of psychic distress, including avoidant and restrictive eating behaviors, increased tension with everyday stressors, tearful episodes, emotional dullness, and occasionally slowed spontaneous movements. She received a tentative diagnosis of psychosis with prevalent negative symptoms and aberrant eating behaviors. As a result, when she was 25 years old, she started working as an employee in a supported job environment. The patient presented several physical comorbidities, including Hashimoto’s thyroiditis (treated with levothyroxine), recurrent episodes of anemia, bone density loss, and amenorrhea secondary to malnutrition. A revised diagnosis of level 2 ASD was determined based on the patient’s medical and neurodevelopmental history, the SCID-5-CV (1, 49), and the RAADS-R (43). Despite a borderline full scale-intelligence quotient (FS-IQ) of 72 as measured with the WAIS-IV (47), comorbid moderate intellectual disability was diagnosed due to prominent impairments in adaptive functioning based on the WHODAS 2.0 (48). Further neuropsychological assessment conducted as part of the routine diagnostic process corroborated the diagnosis, showing an Overall Cognitive Index below the norm, as well as deficits in immediate and delayed prose memory, interference memory, attention shifting, phonemic fluency, verbal comprehension, abstraction abilities, cognitive estimations, visual recognition and discrimination, and praxis (**Table 1**) (51). By the time of her presentation to the Unit of Psychiatry outpatient service, she was not on any psychotropic medications. She exhibited mild obsessive-compulsive symptoms, irritability, anxiety, and depressive thoughts, although not sufficient to diagnose a comorbid major mental health disturbance. Past episodes of acute psychic distress were treated with short-term psychotropic medications, including amisulpride, aripiprazole, cariprazine, and quetiapine, but these attempts never achieved complete symptom remission and often led to the occurrence of adverse effects.

The patient was then prescribed um-PEA 600 mg/day. Hematology and biochemistry tests (i.e., full blood count, electrolytes, renal profile, hepatic profile, bone profile, lipids, thyroid profile), alongside physical examination, were performed and resulted in norm both before initiating the treatment and throughout the whole observation. Substantial clinical stability emerged through the GSI and most subscales of the SCL-90-R (52), as well as in the total anxiety and depression scores of the HADS (53) during the initial 12 weeks of treatment. However, the hostility and sleep subscales of the SCL-90-R (52) slightly worsened (**Tables 2A**, **B**).

At Week 12, better cognitive readiness and work performance were reported according to the patient’s employer, although the total score on the WHODAS 2.0 (48) indicated a global worsening of adaptive functioning, with the Getting along with people domain declining the most (**Table 2C**). She maintained um-PEA for 4 months, with no reported side effects (**Table 2D**). Subsequently, the clinical picture deteriorated due to the occurrence of racing intrusive thoughts and worries, accelerated speech, increased irritability, and disturbed sleeping pattern. This led the patient to seek emergency care, resulting in the initiation of olanzapine at a dose of up to 7.5 mg/day. Concurrently um-PEA supplementation was discontinued.

Despite resulting in good control over observed symptoms, perceived psychic distress got worse by Week 36, as measured using the GSI of the SCL-90-R (52). Consistently, the somatization, obsessive-compulsive, interpersonal sensitivity, depression, anxiety, phobia, and psychoticism subscales of the SCL-90-R (52) worsened, as well as anxiety and depression scores of the HADS (53) (**Tables 2A**, **B**). Adaptive functioning globally improved from Week 12 to Week 24 according to the WHODAS 2.0 (48) (**Table 2C**). Peripheral immune response biomarkers fluctuated throughout the observation period, with decreases in total WBC count, neutrophil/lymphocyte ratio, SII index, IL-8, IL-2Rα, and IL-10 at either Week 12 or Week 24 compared to baseline, most of which rebounded to higher levels by Week 36. Levels of TNF-α, IP10, IFN-γ, ESR, CRP, and monocyte count increased either progressively or abruptly until Week 24 compared to baseline, before generally decreasing by Week 36. IL-1β and IL-6 were stable within normal limits throughout the observation period. Nf-L exhibited a trend toward an increase but remained within normal limits (**Table 3**). The score profile of the first component at the PCA-SERS revealed an almost flat trend from baseline to Week 12, followed by a sharp rise by Week 24, and then a decline. The spectral loadings for the main principal component – accounting for nearly 92% of the total variance – suggest that the significant change observed after the third month was linked to a decrease in the antioxidant response (negative ergothioneine and glutathione bands at 482, 1224 cm^−1^ and 664, 912 cm^−1^, respectively) and an increase in inflammatory response (uric acid bands at 636, 812, 886, and 1135 cm^−1^) (58, 59). This trend was reversed by Week 36 (**Figure 1**). Markers of eCBome modulation included a decrease in serum levels of AEA, 2-AG, OEA, PEA, and EPA-EA from baseline to Week 36. Notably, PEA serum levels sharply dropped during um-PEA treatment and remained persistently lower at Weeks 24 and 36 compared to baseline. Conversely, DHA-EA and 2-DHG serum levels increased from baseline to Week 36 (**Table 3**).

## 3 Discussion

Both autistic patients presented above underwent um-PEA 600 mg/day monotherapy to treat symptoms of psychic distress for the intended 12-week period, which was extended by an additional month, without experiencing any adverse effects necessitating treatment discontinuation. Overall, um-PEA supplementation was associated with either improvement or at least stability of symptoms for the initial 12 weeks of treatment. Interestingly, the first subject experienced partially relapsed symptoms of depression and interpersonal sensitivity after treatment discontinuation, which may have also impacted the patient’s participation in social activities. The second subject exhibited a sudden decline in her clinical condition after four months of treatment, resulting in the discontinuation of um-PEA and the initiation of antipsychotic medication. Changes in both clinical presentations were accompanied by fluctuations in peripheral immune and eCBome biomarkers throughout the entire observation period. Some considerations from the illustrated reports deserve to be highlighted.

Um-PEA 600 mg/day monotherapy in a level 1 autistic subject appeared to alleviate symptoms of psychic distress and their impact on daily living, with partial regression observed after treatment discontinuation. Notably, some improvements persisted a few months after um-PEA suspension, underscoring the need for further studies to fully understand the symptom-specific properties of um-PEA monotherapy across several psychopathological constructs and adaptive functioning domains in level 1 autistic adults. Contrastingly, um-PEA effects were perceived as more nuanced in a level 2 autistic subject with comorbid intellectual disability, being limited to anecdotal improvements in cognitive performance. Additionally, um-PEA was apparently insufficient to prevent the worsening of psychic distress symptoms in the latter subject, such as thought perseverance, increased irritability, and sleep/wake rhythm disturbances. These issues necessitated psychopharmacological intervention, suggesting caution when considering um-PEA monotherapy for level 2/level 3 autistic individuals. This finding seems noteworthy, because prior research has shown that PEA add-on therapy in risperidone-treated autistic children with moderate to high levels of irritability has led to improved behavior, albeit over shorter durations and with higher dosages of the dietary supplement (40). As add-on therapy for autistic adults with substantial or very substantial support needs, um-PEA could serve for enhancing the effectiveness of conventional psychotropic medications, potentially reducing the necessary dosage of treatments that often come with adverse effects (e.g., second-generation antipsychotics, mood stabilizers) (60, 61). Besides, um-PEA add-on may help treat either somatic and autonomic symptoms often associated with ASD (e.g., dental/periodontal issues, bowel disturbances) (62–66), or commonly comorbid immune disorders (e.g., atopic dermatitis) (67, 68).

Individual fluctuations in the peripheral levels of immune biomarkers and PCA-SERS analysis would indicate diverse inflammatory tendencies between the two patients in parallel to um-PEA intake, with the first subject showing a trend toward reduced inflammation, while the second subject maintained a basically stable response. Intriguingly, peripheral levels of proinflammatory cytokines most implicated in ASD pathophysiology (i.e., IL-1β, TNF-α, and IL-6) showed a decreasing or at least unvarying trend in the level 1 autistic patient, while they increased or remained stable in the level 2 autistic patient throughout the treatment (15–18). Nevertheless, only few cytokines exhibited significant peripheral tone variations following um-PEA supplementation, particularly indicating diametral effects on T-cell homeostasis (i.e., IL-2Rα) and anti-inflammatory response (i.e., IL-10), as well as overlapping effects on interferon activation (i.e., IP10). Further studies will be needed to better explain whether and how um-PEA exerts its immunomodulatory functions in autism with different severity degrees.

No remarkable changes in Nf-L levels were detected in either patient, aligning with early sparse evidence on the role of this specific neuroaxonal injury biomarker in ASD (69, 70), possibly due to its high inter-individual variability and still unclear release pattern across neuropsychiatric conditions (71).

Levels of eCBs and NAEs largely varied over the observation period, potentially following um-PEA intake, though yielding contrasting findings between the two individuals. Consistent with um-PEA mechanism of action (31, 32), the first subject exhibited increased eCBs and NAEs levels during its supplementation. Particularly, AEA signaling paralleled to changes in depressive symptoms and social engagement during the entire observation period, coherent with previous clinical and preclinical evidence accounting for increased AEA levels as a plausible mechanism underlying um-PEA effects on these manifestations (36, 72–74). On the other hand, the second subject exhibited mostly stable eCBs and NAEs levels during treatment, except for a keen decrease in initially elevated PEA tone from baseline to Week 12, only partially halting after um-PEA discontinuation.

This reducing pattern observed during um-PEA supplementation could have been caused by either a negative feedback mechanism, or by gut malabsorption related to her malnutrition state, or other individual factors, such as genetics, long-term effects of preterm birth, or autism severity degree, and could underpin the patient’s unchanged or even worsened clinical condition. Given the second patient’s medical history, it is possible to speculate that at the time of her presentation she was exhibiting early symptoms of psychosis recrudescence, which have previously been linked to elevated PEA serum levels (37) and may have precipitated despite um-PEA treatment.

Although the use of robust psychometric tools (48, 52–54) and biological assessments (55–58) would indicate diametrical effects of um-PEA in autistic adults with different severity levels, the exploratory nature and inherent biases of case reports, including self-assessments with limited insight and physiological variability in inflammatory biomarkers or eCBs/NAEs levels independent of um-PEA intake, and the scarcity of existing literature on the subject (40–42), should not be overlooked. Therefore, it is essential to interpret these findings with caution.

Future research involving patient cohorts and in the context of more rigorous clinical trials should systematically investigate the biological underpinnings of um-PEA monotherapy for the treatment of psychic distress among autistic adults. This would help clarify potential correlations between symptom improvement and biomarker fluctuations, thereby parsing um-PEA potential as a putative disease-modifying drug (75). Finally, whether the biobehavioral effects of um-PEA, either as monotherapy or add-on therapy with a safe and tolerable profile, are to be considered age- and level-specific in autism will require further robust investigations involving patients across the entire spectrum.

## Data Availability

The original contributions presented in the study are included in the article/**Supplementary Material.** Further inquiries can be directed to the corresponding author.
