# Supplementary material for: Assessing the biobehavioral effects of ultramicronized-palmitoylethanolamide monotherapy in autistic adults with different severity levels: a report of two cases
Source: Front Psychiatry. 2024 Oct 22;15:1463849. doi: 10.3389/fpsyt.2024.1463849 (PMC11536324; doi:10.3389/fpsyt.2024.1463849)
Supplement: Supplementary file 1 [file Table1.docx]

**Supplementary Material**

Surface enhanced Raman scattering (SERS) spectroscopy of biofluids is a rapid, label-free and non-destructive technique that can quickly reveal molecular fingerprint information about the biochemical composition of a sample [1]. This is done by analysing changes in the SERS spectral profiles, either in comparison to control samples or over time or dosage, rather than focusing on specific biomarkers. It is worth mentioning that due to the complex nature of human serum, with its multitude of molecules, it has been extremely challenging to accurately identify specific molecules based on SERS bands. Despite previous reports indicating that certain vibrational bands were identifiable [2, 3], further analysis is required. Here, the dynamic changes in the features of SERS bands provide an opportunity to uncover the biochemical pathways that may arise from PEA supplementation. Principal components analysis (PCA) was conducted to examine the multivariant trends in the data and identify the key differences in biochemical reaction paths at each time point. In general, the PCA-SERS analysis of serum samples indicates that the major fingerprint observed for the two patients can be related to inflammatory and antioxidant status. The score time profiles of the first three principal components (PCs) of the SERS dataset describe the several relative abundances changes in time of the main spectral features, highlighted in the corresponding spectral loadings (Figure 1).

References

1. Moisoiu, V., S. D. Iancu, A. Stefancu, T. Moisoiu, B. Pardini, M. P. Dragomir, N. Crisan, L. Avram, D. Crisan, I. Andras*, et al.* "Sers liquid biopsy: An emerging tool for medical diagnosis." *Colloids Surf B Biointerfaces* 208 (2021): 112064. 10.1016/j.colsurfb.2021.112064. <https://www.ncbi.nlm.nih.gov/pubmed/34517219>.

2. Premasiri, W. R., J. C. Lee and L. D. Ziegler. "Surface-enhanced raman scattering of whole human blood, blood plasma, and red blood cells: Cellular processes and bioanalytical sensing." *J Phys Chem B* 116 (2012): 9376-86. 10.1021/jp304932g. <https://www.ncbi.nlm.nih.gov/pubmed/22780445>.

3. Fornasaro, S., V. Sergo and A. Bonifacio. "The key role of ergothioneine in label-free surface-enhanced raman scattering spectra of biofluids: A retrospective re-assessment of the literature." *FEBS Lett* 596 (2022): 1348-55. 10.1002/1873-3468.14312. <https://www.ncbi.nlm.nih.gov/pubmed/35152417>.
